# Multiple-stable anisotropic magnetoresistance memory in antiferromagnetic MnTe

**DOI:** 10.1038/ncomms11623

**Published:** 2016-06-09

**Authors:** D. Kriegner, K. Výborný, K. Olejník, H. Reichlová, V. Novák, X. Marti, J. Gazquez, V. Saidl, P. Němec, V. V. Volobuev, G. Springholz, V. Holý, T. Jungwirth

**Affiliations:** 1 Charles University in Prague, Ke Karlovu 3, Praha 2 121 16, Czech Republic; 2 Institute of Physics, Academy of Science of the Czech Republic, Cukrovarnická 10, Praha 6 162 00, Czech Republic; 3 Institut de Ciència de Materials de Barcelona ICMAB, Consejo Superior de Investigaciones Científicas CSIC, Campus UAB, Bellaterra, Catalonia 08193, Spain; 4 Institute of Semiconductor and Solid State Physics, Johannes Kepler University Linz, Altenbergerstraβe. 69, Linz 4040, Austria; 5 National Technical University, ‘Kharkiv Polytechnic Institute', Frunze Str. 21, Kharkiv 61002, Ukraine; 6 School of Physics and Astronomy, University of Nottingham, Nottingham NG7 2RD, UK

## Abstract

Commercial magnetic memories rely on the bistability of ordered spins in ferromagnetic materials. Recently, experimental bistable memories have been realized using fully compensated antiferromagnetic metals. Here we demonstrate a multiple-stable memory device in epitaxial MnTe, an antiferromagnetic counterpart of common II–VI semiconductors. Favourable micromagnetic characteristics of MnTe allow us to demonstrate a smoothly varying zero-field antiferromagnetic anisotropic magnetoresistance (AMR) with a harmonic angular dependence on the writing magnetic field angle, analogous to ferromagnets. The continuously varying AMR provides means for the electrical read-out of multiple-stable antiferromagnetic memory states, which we set by heat-assisted magneto-recording and by changing the writing field direction. The multiple stability in our memory is ascribed to different distributions of domains with the Néel vector aligned along one of the three magnetic easy axes. The robustness against strong magnetic field perturbations combined with the multiple stability of the magnetic memory states are unique properties of antiferromagnets.

## Abstract (web summary)

Contrary to ferromagnets, antiferromagnets possess no net magnetic moment, which has limited their applicability as magnetic memory media. Here, the authors demonstrate a heat-assisted multiple-stable memory based on epitaxial thin films of antiferromagnet MnTe with three-fold symmetric anisotropy.

## Graphical abstract

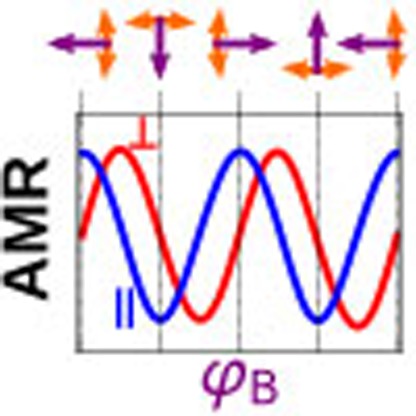

A common perception assumes that magnetic memories require ferromagnetic materials with a non-zero net magnetic moment. However, it has been recently proposed that compensated antiferromagnets with a zero net moment may represent a viable alternative to ferromagnets^1^,^2^,^3^,^4^,^5^. So far, experimental research has focused on bistable memories in antiferromagnetic metals^6^,^7^,^8^,^9^,^10^. Switching between two memory states in metallic antiferromagnets has been demonstrated in FeRh by taking advantage of its high-temperature ferromagnetic phase^8^,^9^. Similar effects were shown in thin-film IrMn tunnelling devices utilizing the exchange-spring with an adjacent ferromagnetic layer^6^ or field-cooling for switching the antiferromagnetic moments^7^. In CuMnAs, current induced torques were used to switch the antiferromagnet without an auxiliary ferromagnetic element and without a magnetic field^10^. The two distinct stable states of these antiferromagnetic-metal memories showed distinct resistances which allowed for their electrical read-out. The physical mechanism of the read-out is ascribed in these devices to the antiferromagnetic anisotropic magnetoresistance (AMR). The AMR interpretation of the electrical read-out signals appeals to the general principle formulated by Louis Néel in his Nobel lecture that: “Effects in antiferromagnets depending on the square of the spontaneous magnetisation should show the same variation as in ferromagnetic substances” (http://www.nobelprize.org/nobel_prizes/physics/laureates/1970/neel-lecture.pdf)^11^. Indeed, AMR is an even function of the magnetisation and in principle should therefore be equally present in antiferromagnets as in ferromagnets.

In ferromagnets, where AMR is well studied^12^, a common characteristic feature is its harmonic 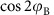 and 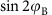 dependence on the angle 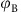 between the current and the applied saturating magnetic field when measured with the longitudinal and transverse voltage probes, respectively. Controlling the Néel order by external magnetic fields is, however, significantly more difficult, which has hindered the detection of the characteristic angular dependence of the AMR in antiferromagnets. For a more detailed explanation of the AMR see the Supplementary Note 1.

Below we present measurements of the harmonic-function AMR in our Hall bar devices made in antiferromagnetic semiconductor MnTe and by this directly illustrate the applicability of the above Néel's principle on this basic spintronic phenomenon. Moreover, unlike the previously studied bistable antiferromagnetic-metal memories, our MnTe devices allow us to set multiple-stable antiferromagnetic memory states by heat-assisted magneto-recording. When removing the writing magnetic fields sufficiently below the Néel temperature, the harmonic AMR-like signal is still preserved in the zero-field read-out traces. As expected for antiferromagnets, these multiple-stable states are not erased by even strong magnetic-field perturbations as we also demonstrate below. The robustness and the possibility of setting a continuum of states makes them promising candidates for highly stable memory states.

## Results

### Characterization of MnTe thin films

**Figure 1:**
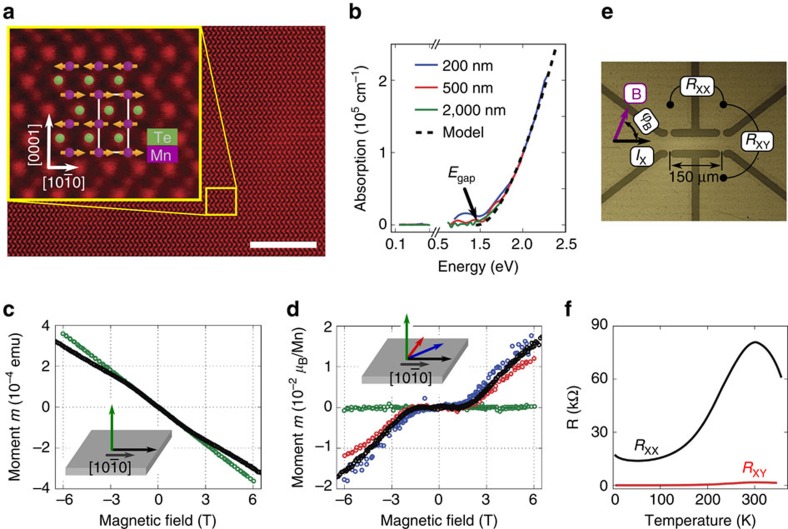
MnTe thin films properties. (**a**) Cross-sectional HAADF image taken along the 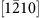 zone axis resolving the individual atomic columns of Mn (magenta) and Te (green). The inset shows the atomic positions including the Mn magnetic moments in the ordered antiferromagnetic state and the unit cell size is indicated by a white line. Scale bar, 5 nm. (**b**) Absorption coefficient of MnTe in the mid-infrared and visible spectral range extracted from transmission measurements for three different film thicknesses. (**c**) SQUID magnetic field sweeps for in-plane and out-of-plane field directions at 5 K. The diamagnetism of the substrate is dominating the behaviour. (**d**) SQUID magnetic field sweeps for several indicated field directions at 5 K with the diamagnetism of the out-of-plane measurement subtracted from all the measured traces. (**e**) Micrograph of the Hall bar geometry indicating the longitudinal (*R*_XX_) and transverse (*R*_XY_) resistances together with the definition of the magnetic field angle 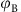. (**f**) Temperature-dependent longitudinal and transverse resistance measurement showing a peak near the transition temperature in the longitudinal resistance.

We use MnTe thin films grown epitaxially on single crystalline InP substrates with (111) oriented surface by molecular beam epitaxy. Before discussing our main results we first show structural, optical, magnetic and electrical characterization of the samples. The structural quality of our epilayers is evidenced in Fig. 1a which shows a high-resolution high-angle annular dark-field (HAADF) image of individual atomic Te and Mn columns as bright and dimmer spots, respectively. In contrast to most thin-film MnTe studies^13^,^14^,^15^, we obtain the hexagonal NiAs bulk structure^16^ (*α*-MnTe) owing to the good lattice matching to the substrate. X-ray diffraction measurements (Supplementary Fig. 1) show that this is the only phase present in our films and together with HAADF images of the interface (Supplementary Fig. 2) confirm the epitaxial growth with *c*-planes aligned parallel to the substrate surface. From X-ray diffraction reciprocal space maps for the 50-nm-thick epilayer used in the transport experiments we conclude that the film is relaxed forming a mosaic structure with a block size of 25±5 nm determined by fitting a mosaic block model^17^. The relative misorientation of the blocks is small with a Gaussian distribution with s.d. of 0.25±0.1°. (For more details on the X-ray characterization see Supplementary Note 2 and Supplementary Fig. 3.)

The semiconducting electronic structure of our *α*-MnTe films is confirmed by optical absorption measurements (Supplementary Fig. 4) in the visible and mid-infrared range on epilayers deposited on a transparent SrF_2_(111) substrate. X-ray diffraction and HAADF images confirm the equal quality of these films to the ones grown on InP (Supplementary Figs 2 and 3). We performed measurements for layers of different thicknesses, which all show the same onset of the absorption shown in Fig. 1b. From this we infer an indirect bandgap of 1.46±0.10 eV in agreement with previously reported bulk values^16^,^18^,^19^.

Below the Néel temperature, which is 310 K (ref. 20) in bulk *α*-MnTe, the magnetic structure consists of ferromagnetically ordered Mn-planes which are antiferromagnetically stacked along the *c*-direction^20^. Neutron diffraction experiments^21^,^22^ and susceptibility measurements^23^ (see also Supplementary Fig. 1b) found that the moments lie within the Mn-planes, as indicated in Fig. 1a. The moderate Néel temperature and the expected weak in-plane magneto-crystalline anisotropy in the hexagonal structure suggest that significant magnetic moment reconfigurations might be achievable at moderate applied magnetic fields. Low-temperature magnetization data measured by the superconducting quantum interference device (SQUID) and shown in Fig. 1c,d confirm these expectations. Consistent with the antiferromagnetic order, we observe a zero remanent moment and even at high fields, the magnetization per Mn atom is only a minor fraction of the Mn magnetic moment of 4.8*μ*_B_ (refs 21, 22). The moderate exchange energy (Néel temperature) and expected weak in-plane anisotropies are reflected in the onset of a sizable magnetic moment at a moderate threshold field of ∼2 T in the in-plane magnetic field sweeps.

As shown in Supplementary Note 3 including Supplementary Figs 5 and 6, the threshold which may be associated with the spin-flop field in the domains moves to lower fields when approaching the Néel temperature. Because of the hexagonal crystal structure and antiferromagnetic moments oriented in the *c*-plane, the system can break up into three types of domains in which the Néel vector points along one of the three high-symmetry directions^23^. In *α*-MnTe single crystals, it was demonstrated that the distribution of these domains can be rearranged by cooling the system through the antiferromagnetic transition in an applied magnetic field^23^. All these favourable magnetic characteristics are the basis of our experiments in the MnTe memory devices presented below.

Transport measurements performed in a Hall bar geometry shown in Fig. 1e,f complete the basic characterization of our thin-film *α*-MnTe samples. The temperature-dependent zero-field longitudinal resistance shows a peak near the Néel temperature associated with critical scattering off spin fluctuations^24^. Note that critical anomalies in our films are consistently observed also in the susceptibility^23^ and lattice parameter measurements (see Supplementary Figs 1 and 7). The transverse resistance should vanish at zero field in both the paramagnetic phase above the Néel temperature and in the antiferromagnetic phase with no preferred direction among the easy axes set during the zero-field-cooling. The weak transverse resistance signal seen in Fig. 1f is ascribed to an unintentional asymmetry of the Hall bar transverse contacts and the resulting small admixture of the longitudinal signal. From the Hall effect measurements, we obtained low-temperature hole density of *P*=6 × 10^18^ cm^−3^ due to unintentional doping in our film and corresponding hole mobility of *μ*=43 cm^2^ V^−1^ s^−1^.

### Antiferromagnetic anisotropic magnetoresistance memory

**Figure 2:**
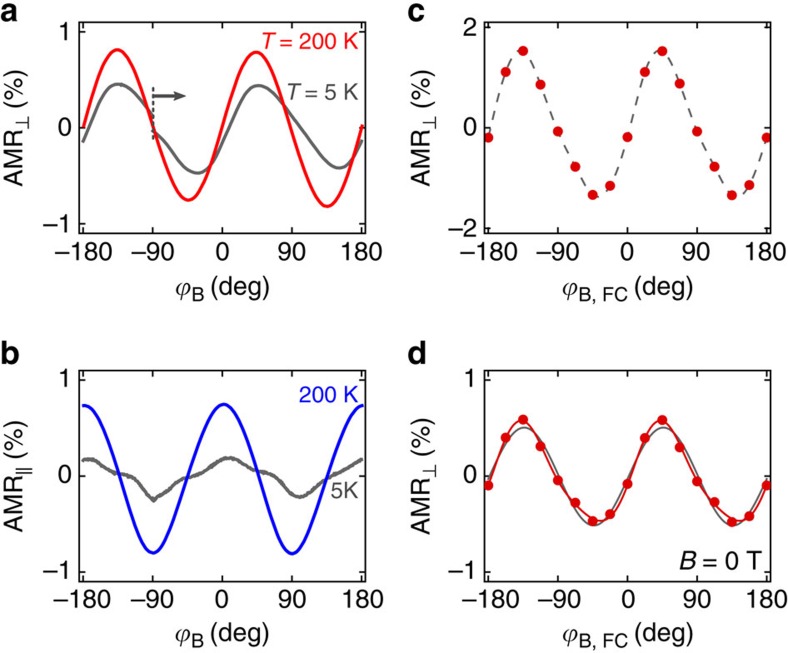
Antiferromagnetic AMR and multiple-stable memory. (**a**,**b**) Transverse (red) and longitudinal (blue) AMR measurements at 200 K and rotating in-plane 2 T field. Analogous measurements at 5 K are shown in grey. Arrow in **a** indicates the initial angle and the direction of rotation. (**c**) Transverse AMR measured at 200 K after cooling from 350 K in a magnetic field (*B*_FC_=2 T) applied at an angle 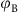
_,FC_ and with the field kept on. The dashed line is a guide to the eye. (**d**) Zero-field transverse AMR obtained after field-cooling (*B*_FC_=2 T) down to 5 K then removing the field and taking zero-field resistance measurements at 200 K. The grey line is a 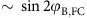 least squares fit which fails to describe the details of the angular variation in the experimental data. On the other hand, the red line shows the least square fit of the multi-domain model calculations, which accurately reproduce the angular variation of the experimental data.

We now proceed to the discussion of the antiferromagnetic AMR and memory functionalities in our MnTe devices. In Fig. 2a,b, we plot the transverse and longitudinal AMRs, defined as 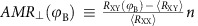 and 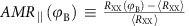, where 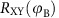 and 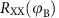 are the transverse and longitudinal resistances indicated in Fig. 1e, 〈〉 denotes averaging over all angles 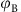 between the magnetic field and current, and *n* is the aspect ratio of our Hall bar. Measurements in Fig. 2a,b are performed at constant temperature of 200 K, sufficiently below the Néel temperature, and in a rotating 2 T field. The curves show a harmonic 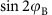 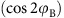 dependence on the field-angle and the amplitudes of *AMR*_⊥_ and *AMR*_||_ scale with the Hall bar aspect ratio, that is, corresponding curves in Fig. 2a,b have the same amplitude. This phenomenology is reminiscent of common non-crystalline AMR traces in ferromagnets in applied saturating magnetic fields, where the ratio of the longitudinal and transversal AMR amplitudes is also unity (see Supplementary Note 1). Note that a crystalline AMR contribution, due to an additional dependence of the resistance on the angle between magnetic moments and crystal axes, is negligible at 200 K (Supplementary Note 3).

For comparison, we show in Fig. 2a,b also 2 T AMR curves measured at a low temperature (5 K). In contrast to the data at 200 K, the corresponding traces are anharmonic, show history dependence, have smaller magnitudes and the amplitudes of *AMR*_||_ and *AMR*_⊥_ are significantly different. This is a result of the stiffening of the MnTe antiferromagnet at 5 K, where the 2 T field causes only partial reorientation of the spin-axes in the domains around their zero-field direction, reminiscent of ferromagnets in weak fields below the saturation field. The *AMR*_||_ and *AMR*_⊥_ signals therefore differ in their amplitude and depend on the previous field-cooling protocol. Consistently, Supplementary Fig. 8 shows a more systematic study of the ratio of the measured *AMR*_||_ to *AMR*_⊥_ amplitude whose deviation from unity and history dependence is largest at low fields and low temperatures. Together with a non-negligible contribution of the crystalline AMR this results in a complicated anharmonic shape of the longitudinal AMR trace at 5 K and 2 T shown in Fig. 2a,b.

In Fig. 2c, we show AMR measurements in which, for each point, we first heated the sample above the Néel temperature (to 350 K) and then field cooled (with *B*_FC_) down to 200 K in a 2 T field of a fixed angle 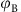,_FC_ and measured the corresponding resistance with the field on. To obtain the data shown in Fig. 2d, we continued with the field-cooling down to 5 K then removed the field and took zero-field resistance measurements again at 200 K. Corresponding data for other read-out temperatures are shown in Supplementary Fig. 9a. Remarkably, we observe similar AMR traces in the two panels only the amplitude of the zero-field AMR in Fig. 2d is about a factor 2 smaller than in Fig. 2c.

Note that a 200 K zero-field AMR of a comparable amplitude to the one seen in Fig. 2d is also obtained when field-cooling from 350 K down to only 200 K. Similar results can also be obtained with other heat-assisted magneto-recording protocols where the Néel temperature is not crossed. For instance, it is also possible to set a stable zero-field AMR trace of the form of Fig. 2d, only with a factor 2 smaller amplitude, by field-cooling from 200 K down to low temperatures and then performing the zero-field read-out measurement back at 200 K (see Supplementary Fig. 10). This is consistent with the ability, seen in Fig. 2a,b, to control the antiferromagnetic state by the 2 T field even if below, but not too far from the Néel temperature. Crossing the Néel temperature in the heat-assisted magneto-recording, therefore, helps the efficiency of the writing process, however, it is not necessary. We emphasize that Fig. 2 not only demonstrates a continuous harmonic-function AMR in an antiferromagnet but it also shows a multiple stability of states in our MnTe memory device.

### Stability of the memory states

**Figure 3:**
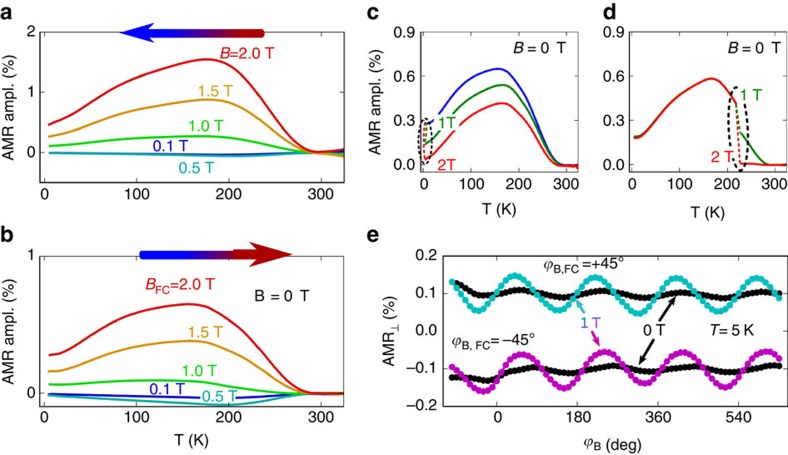
Dependence on writing field and robustness against field perturbations. (**a**) Temperature and writing field-strength dependence of the AMR amplitude for the temperature down-sweep with the writing field kept on. (**b**) Temperature and writing field-strength dependence of the zero-field AMR amplitude for the temperature up-sweep. (**c**) Zero-field AMR amplitudes for the temperature up-sweep after exposing the memory at 5 K to 1 T (green) and 2 T (red) perturbing fields rotated both in-plane and out-of-plane. The blue curve represents the unperturbed reference measurement. A field-cooling strength of *B*_FC_=2 T was used in all three cases. (**d**) Same as **c** for the field perturbations applied at 200 K. (**e**) The stability of two memory states set by the heat-assisted magneto-recording from 350 K with a 2 T writing field applied at angles 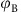
_,FC_=±45° tested at 5 K by a rotating 1 T field. At every angle, 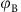 of the perturbing 1 T field, magenta/turquoise curves correspond to the read-out resistance measurements with the field on, while black lines are obtained after removing the perturbing field at each 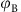.

Next we explore how the amplitude of the memory read-out signal depends on the strength of the writing field and test the limits for erasing the multiple-stable states by turning the magnetic field back on. In Fig. 3a, we plot the AMR amplitude, defined as 
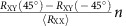
, obtained when setting the states by field-cooling at 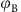
_,FC_=±45° from 350 K down to 5 K and measuring the resistance during the temperature down-sweep with the field on. Figure 3b shows zero-field AMR amplitudes measured during the subsequent up-sweep in temperature. The variation of the signal is associated with the dependence of the AMR coefficients on temperature (see Supplementary Note 1). The AMR disappears near the Néel temperature and at low temperatures the AMR is again reduced, presumably due to the suppressed contribution from magnon and phonon scattering. This leads to a peak of the AMR amplitude in Fig. 3a,b at intermediate temperatures.

For the range of writing field magnitudes from 0.5 to 2 T, we were able to set the multiple-stable memory states with the zero-field AMR of the harmonic form seen in Fig. 2d. The signal disappears for all traces at ∼285 K, which approximately coincides with the Néel temperature of thin *α*-MnTe films^25^ and is just ∼10 K lower than the broad peak in the longitudinal resistivity shown in Fig. 1d. This confirms the antiferromagnetic origin of the read-out signal. The amplitude of the AMR signal scales with the magnitude of the writing field, apart from the region between 0.5 and 1 T, where the AMR signal changes sign, and does not saturate at the maximum applied writing field of 2 T. Before discussing the origin of these features we complete in Fig. 3c,d the description of experimental data, namely of the measurements testing the robustness of the multiple-stable memory states under magnetic-field perturbations.

In Fig. 3c, we replot the zero-field AMR amplitude obtained from the temperature up-sweeps after setting the states in 2 T writing fields. We compare the trace with analogous measurements which only differ in an additional magnetic field exposure of the memory at 5 K after the writing and before starting the temperature up-sweep read-out measurement. The additional exposure comprises application of rotating in-plane and out-of-plane magnetic fields of a 1 or 2 T magnitude. We see that neither of these two magnitudes of the disturbing field applied at any angle is sufficient to fully erase the memory. After the exposure, the multiple-stable states maintain their characteristic harmonic AMR form of the read-out signal (see Supplementary Note 4 and Supplementary Fig. 9) with only the amplitude being partially reduced, as shown in Fig. 3c.

We performed similar attempts to erase our antiferromagnetic memory at 200 K. The results, shown in Fig. 3d, illustrate that at this elevated temperature a magnetic field of 2 T is sufficient to fully erase the memory states. This is consistent with measurements in the rotating field of 2 T at 200 K shown in Fig. 2a,b, where the observed AMR traces had a character of AMR in ferromagnets under saturating magnetic fields. A field of 1 T, on the other hand, is not sufficient to erase the memory at 200 K; it only reduces the amplitude of the read-out signal, as seen in Fig. 3d.

In Fig. 3e, we illustrate in more detail the stability of our antiferromagnetic memory in fields, which are insufficient to erase it. We explore how the states set by cooling in writing fields of 2 T applied at angles 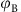
_,FC_=±45°, corresponding to the extrema in the *AMR*_⊥_ read-out signal, are disturbed at 5 K by a field of 1 T rotating in the sample plane. By taking the resistance measurements with the field on, we observe a partial reorientation of the antiferromagnetic spin-axis, reflected in the varying resistance signal. The variations are, however, smaller than the difference between the zero-field resistances of the two extrema and according to our modelling presented below correspond to only ∼3° rotation of the Néel vector and concomitant canting of the antiferromagnetic moments by ∼1°. Moreover, if at each given angle of the disturbing magnetic field we remove the field and repeat the resistance measurement, we see that the original zero-field state almost fully recovers. Supplementary Fig. 11 shows the measurements discussed in Fig. 3e but for applied disturbing fields of 0.5 and 2 T. The 0.5 T field is insufficient to cause any significant changes of the AMR signal neither with the disturbing field on nor after turning the 0.5 T field off. On the other hand, the 2 T disturbing field causes much stronger transient changes of the AMR as compared with the 1 T field and significant permanent changes of the AMR signal remain even after turning the 2 T field off. This is consistent with the measurements shown in Fig. 3c.

### Modelling of the zero-field AMR signal

**Figure 4:**
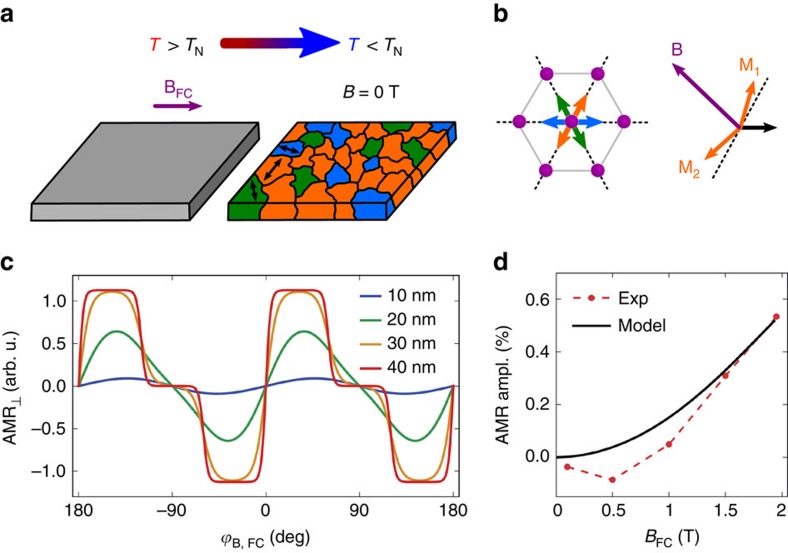
Modelling of the multiple-stable states. (**a**) Schematics of setting the multiple-stable states, each with a distinct domain distribution, by the heat-assisted magneto-recording across the Néel temperature. The colour of the domains after the field-cooling indicates the different Néel vector orientations in the various domains and uses the same colour coding as arrows in **b**. (**b**) Schematics of the orientation of the three in-plane easy axes in hexagonal *α*-MnTe with respect to the Mn atoms (purple circles) together with a sketch of the two antiferromagnetic spin sublattices **M**_1,2_ which tend to align perpendicular to the applied magnetic field **B** and to cant towards the field. Black dashed lines indicate the easy axes orientations. (**c**) Modelling of the distribution of the three domains in the multiple-stable memory states and of the corresponding transverse AMR signal for different size of the domains. (**d**) Comparison of the model prediction (black line) and experimental dependence at 200 K (red) of the read-out AMR amplitude on the strength of the writing magnetic field.

We now proceed to the discussion of the origin of the observed multiple-stable aniferromagnetic memory states and the corresponding zero-field harmonic-function AMR. When cooling the system in an applied magnetic field, the domain distribution freezes at temperatures sufficiently below the Néel temperature, as illustrated in Fig. 4a. The freezing occurs when the thermal fluctuations no longer allow the different domains to flip their spin-axis from one to another metastable direction.

The writing magnetic field applied at a certain angle during the cooling makes the easy axis closest to the field normal more favourable (see sketches in Fig. 4b). Because of the finite temperature, however, the other two easy axis directions can also be populated. To model the population of these three types of domains, we determine their energy within the Stoner–Wohlfarth model for antiferromagnets^26^. This involves finding the metastable orientations of the antiferromagnetic moments in each of the three easy-axis domains for a given applied field during the field-cooling. With these orientations we calculate the energy of the three types of domains and obtain the corresponding relative occupation of the domains from the Boltzmann statistics. From the calculated domain occupation and the angles between the easy axes and the current direction we calculate the corresponding net zero-field AMR. Details about the applied model are given in the Supplementary Note 5 and Supplementary Fig. 12.

The energy of the system comprises the exchange energy, the magnetic anisotropy energy and the Zeeman energy. Their relative importance for the domain population depends on the domain size and applied magnetic field. When the Zeeman term dominates, that is, for very large magnetic fields or domain sizes, one of the three easy directions would be strongly favoured within a 60° interval of the writing field angles, and the other directions for the other respective 60° intervals. The read-out AMR signal in this case would take the form of a step-like trace with three distinct memory states. Upon reducing the Zeeman contribution, the AMR trace undergoes a transition into a continuous function whose amplitude gradually decreases and whose shape approaches the harmonic 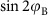
_,FC_ function of the writing field angle (see Fig. 4c). Since our measured signals plotted in Fig. 2d show a weak but clearly detectable deviation from the harmonic 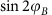
_,FC_ form, we can infer the typical domain size from fitting the model to the measured data.

To reduce the number of free parameters in the calculations, the exchange interaction is estimated from the Néel temperature and we use the magnetic moment found in neutron diffraction experiments^21^,^22^. The anisotropy energy was inferred assuming a spin-flop field of 2 T. The remaining free parameters for the calculation of the zero-field AMR traces are the domain size, the AMR amplitude in the single domain state and the direction of the three easy axes. Note that the shape of the domains is not relevant in this model since the contribution from domain walls is neglected and no size distribution of the domains is considered. We use the diameter of spheres as indicative parameter in our model, since spheres were also successfully used in the mosaic block model described earlier. The least squares optimization procedure is used to fit the experimental AMR data.

As seen in Fig. 2d, our model reproduces well the experimental behaviour. From the fitting we obtain that all the three domains each with the antiferromagnetic moments aligned along one of the three different easy axes are at least partially occupied with the highest inequality of 2:1:1 and 2:2:1 for the cases when the writing field is perpendicular or parallel to one of the three easy axes, respectively. The easy orientation of the moments is found to be along 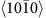 directions as indicated in Figs 1a and 4b. The saturated AMR amplitude is found to be 1.3±0.2%, similar to the experimental AMR amplitude measured during the field-cooling which is expected to be as close as possible to the saturation case. The domain diameter we find in the fitting is 20±5 nm, which is in a good agreement with the mosaic block size obtained from the X-ray diffraction results. From this we conclude that the magnetic domain size is determined by the structural block size and does not change significantly with the writing conditions. Supplementary Table 1 summarises our model parameters.

Under the assumption of a constant domain size, the model also yields the dependence of the amplitude of the zero-field read-out AMR signal on the strength of the writing field. As shown in Fig. 4d, the model predicts an initial quadratic increase of the read-out AMR signal followed by a tendency towards saturation at higher fields. It broadly agrees with the measured data apart from the negative AMR signal seen in the experiment at low writing fields. Note that for this comparison no additional adjustable parameter is introduced in our model and we use the same model parameters as obtained for the fit in Fig. 2d.

The negative AMR signal observed at low fields in Fig. 4d could be attributed to uncompensated moments at domain walls not included in our model. Although the detailed magnetic structure of domain boundaries is unknown they are likely to contain uncompensated moments. While the compensated moments within the antiferromagnetic domains tend to align perpendicular to the applied field, these uncompensated moments prefer a parallel alignment with the field. The uncompensated moments lead to a linear gain in the Zeeman energy which competes with the canting of the spin sublattices in the antiferromagnetic domains whose energy scales quadratically with the applied magnetic field. At low fields, the Zeeman energy due to the uncompensated moments dominates which explains the opposite AMR sign as compared with the high writing field regime dominated by the antiferromagnetic moments.

## Discussion

We have reported spintronic memory functionalities in an antiferromagnetic counterpart of common II–VI compound semiconductors. Favourable magnetic characteristics of our *α*-MnTe epilayers allowed us to evidence the direct analogy between AMR in antiferromagnets and ferromagnets, both in its basic phenomenology and in the utility as an electrical detection tool of the ordered magnetic moments. Our work also highlights the unique potential of antiferromagnets for spintronics. In our MnTe devices, we demonstrated that the harmonic-function AMR persists even after removing the setting magnetic field and that the multiple-stable memory states cannot be erased by strong magnetic field perturbations when sufficiently below the antiferromagnetic transition temperature. These magnetic memory characteristics are unprecedented in ferromagnets. One of the areas of potential applications of antiferromagnetic multiple-stable memories is in electronic variants of neural networks^27^. Here the multi-level variation of a synapsis combined with the non-volatility could serve as a basic for realizing the analogues of neuromorphic systems.

## Methods

### Sample preparation

*α*-MnTe was grown by molecular beam epitaxy on InP(111)A, that is, In-terminated surface, and SrF_2_(111) substrates using elemental Mn and Te sources and substrates temperatures in the range of 370 to 450 °C. Two-dimensional growth was achieved in both cases as judged from the streaked RHEED patterns observed during growth. The orientation of our layers on the substrate is (0001)[10
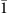
0]_MnTe_|| (111)[11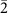]_sub_. For structural characterization we carried out cross-sectional scanning transmission electron microscopy (STEM) in the high-angle annular dark-field (HAADF) imaging mode and X-ray diffraction studies with CuK*α*_1_ radiation. STEM-HAADF images were acquired with a NION UltraSTEM, equipped with a NION aberration corrector and operated at 100 kV. In the images the intensity scales approximately with the atomic number (Z) squared^28^. Cross-sectional STEM specimens were prepared using a FEI Nova 200 Dual-Beam SEM/FIB focused ion beam.

### Magnetometry

Measurements were performed on 2,000-nm-thick MnTe films with area of ∼11 mm^2^ and 500-*μ*m-thick InP substrate in a Quantum Design SQUID magnetometer using reciprocating sample option for increased measurement sensitivity. Magnetic-field sweeps were recorded for various crystallographic directions, while sweeping from negative to positive and vice versa showing no hysteresis. From all magnetic field sweeps, a negative slope obtained from the fit to the hard-axis out-of-plane sweep seen in the inset in Fig. 1c was subtracted to correct for signal coming primarily from the diamagnetic substrate. Temperature-dependent susceptibility measurements were taken in magnetic field of 0.5 T.

### Optics

The transmission spectra of MnTe thin films grown on transparent SrF_2_ were measured in the visible (400–1,100 nm) and mid-infrared (2,000–15,000 nm) range for film thicknesses of 200, 500 and 2,000 nm. Measurements were performed at *T*=300 K. The SrF_2_ substrate is transparent in the full visible spectral range and up to 10 μm in the infrared (Supplementary Fig. 4). Fabry–Pérot oscillations due to the finite film thickness are observed in the transmission data at wavelength above the onset of the strong absorption. The thickness-independent absorption coefficient and thus bandgap properties were determined by fitting the transmission spectra with an analytical expression^29^ for the transmission of a thin absorbing film on a transparent substrate.

### Processing and transport measurements

For transport measurements Hall bars were defined in 50-nm-thick films grown on InP along the [10
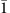
0] direction by electron-beam lithography and wet etching using a H_3_PO_4_/H_2_O_2_/H_2_O mixture as etchant. Contacts were made by soldering with pure In and four-point measurement technique was employed to avoid possible contribution of contact resistances. Complementary Corbino-disk devices were fabricated by deposition of Au rings. The transport measurements were performed in an Oxford Instruments vector field cryostat capable of generating fields up to 2 T in arbitrary directions and up to 6 T in the current direction. A constant DC current density of 2,000 A cm^−2^ was applied during all measurements. For the measurements of *AMR*_||_ in Fig. 2b, a temperature stabilization better than 0.1 K was performed while for measurements of *AMR*_⊥_ shown in Fig. 2c,d the precise temperature stabilization is not required and these data were extracted from temperature sweeps.

## Additional information

**How to cite this article:** Kriegner, D. *et al*. Multiple-stable anisotropic magnetoresistance memory in antiferromagnetic MnTe. *Nat. Commun.* 7:11623 doi: 10.1038/ncomms11623 (2016).

## Supplementary Material

Supplementary Information Supplementary Figures 1-12, Supplementary Table 1, Supplementary Notes 1-5 and Supplementary References

Peer Review file

## References

[b1] Shick A. B., Khmelevskyi S., Mryasov O. N., Wunderlich J. & Jungwirth T. Spin-orbit coupling induced anisotropy effects in bimetallic antiferromagnets: a route towards antiferromagnetic spintronics. Phys. Rev. B 81, 212409 (2010).

[b2] MacDonald A. H. & Tsoi M. Antiferromagnetic metal spintronics. Philos. Trans. A. Math. Phys. Eng. Sci. 369, 3098–3114 (2011). 21727116 10.1098/rsta.2011.0014

[b3] Duine R. Spintronics an alternating alternative. Nat. Mater. 10, 344–345 (2011). 21505467 10.1038/nmat3015

[b4] Gomonay E. V. & Loktev V. M. Spintronics of antiferromagnetic systems. Low Temp. Phys. 40, 17–35 (2014).

[b5] Jungwirth T., Marti X., Wadley P. & Wunderlich J. Antiferromagnetic spintronics. Nat. Nanotechnol. 11, 231–241 (2016). 26936817 10.1038/nnano.2016.18

[b6] Park B. G. . A spin-valve-like magnetoresistance of an antiferromagnet-based tunnel junction. Nat. Mater. 10, 347–351 (2011). 21399629 10.1038/nmat2983

[b7] Petti D. . Storing magnetic information in IrMn/MgO/Ta tunnel junctions via field-cooling. Appl. Phys. Lett. 102, 192404 (2013).

[b8] Marti X. . Room-temperature antiferromagnetic memory resistor. Nat. Mater. 13, 367–374 (2014). 24464243 10.1038/nmat3861

[b9] Moriyama T. . Sequential write-read operations in FeRh antiferromagnetic memory. Appl. Phys. Lett. 107, 122403 (2015).

[b10] Wadley P. . Electrical switching of an antiferromagnet. Science 351, 587–590 (2016). 26841431 10.1126/science.aab1031

[b11] Zhou Y. Physics, 1963–1970 Elsevier Science (2013).

[b12] McGuire T. & Potter R. Anisotropic magnetoresistance in ferromagnetic 3d alloys. IEEE Trans. Magn. 11, 1018–1038 (1975).

[b13] Akinaga H., Ando K., Abe T. & Yoshida S. Control of the crystal orientation of zinc-blende MnTe epitaxial films grown on GaAs. J. Appl. Phys. 74, 746–748 (1993).

[b14] Janik E. . Structural properties of cubic MnTe layers grown by MBE. Thin Solid Films 267, 74–78 (1995).

[b15] Hennion B., Szuszkiewicz W., Dynowska E., Janik E. & Wojtowicz T. Spin-wave measurements on MBE-grown zinc-blende structure MnTe by inelastic neutron scattering. Phys. Rev. B 66, 224426 (2002).

[b16] Allen J. W., Lucovsky G. & Mikkelsen J. C. Optical properties and electronic structure of crossroads material MnTe. Solid State Commun. 24, 367–370 (1977).

[b17] Pietsch U., Holý V. & Baumbach T. High-Resolution X-Ray Scattering 2nd edn, ch. 8, 205–233 Springer-Verlag, New York, USA (2004).

[b18] Zanmarchi G. Optical measurements on the antiferromagnetic semiconductor MnTe. J. Phys. Chem. Solids 28, 2123–2130 (1967).

[b19] Ferrer-Roca C., Segura A., Reig C. & Muñoz V. Temperature and pressure dependence of the optical absorption in hexagonal MnTe. Phys. Rev. B 61, 13679–13686 (2000).

[b20] Madelung O., Rössler U. & Schulz M. (Eds) MnTe: Crystal Structure, Physical Properties Vol. 41D, SpringerMaterials, Springer-Verlag, Berlin, Heidelberg (2000).

[b21] Kunitomi N., Hamaguchi Y. & Anzai S. Neutron diffraction study on manganese telluride. J. Phys. Paris 25, 568–574 (1964).

[b22] Szuszkiewicz W., Hennion B., Witkowska B., Usakowska E. & Mycielski A. Neutron scattering study of structural and magnetic properties of hexagonal MnTe. Phys. Stat. Sol. C 2, 1141–1146 (2005).

[b23] Komatsubara T., Murakami M. & Hirahara E. Magnetic properties of manganese telluride single crystals. J. Phys. Soc. Jpn. 18, 356–364 (1963).

[b24] Magnin Y. & Diep H. T. Monte Carlo study of magnetic resistivity in semiconducting MnTe. Phys. Rev. B 85, 184413 (2012).

[b25] Przedziecka E. . Preparation and characterization of hexagonal MnTe and ZnO layers. Phys. Stat. Sol. C 2, 1218–1223 (2005).

[b26] Bogdanov A. N. & Dragunov I. E. Metastable states, spin-reorientation transitions, and domain structures in planar hexagonal antiferromagnets. Low Temp. Phys. 24, 852–857 (1998).

[b27] Locatelli N. . Spintronic devices as key elements for energy-efficient neuroinspired architectures. In Design, Automation & Test in Europe Conference & Exhibition (DATE), 994–999Institute of Electrical and Electronics Engineers (IEEE) (2015).

[b28] Nellist P. & Pennycook S. Incoherent imaging using dynamically scattered coherent electrons. Ultramicroscopy 78, 111–124 (1999).

[b29] Swanepoel R. Determination of the thickness and optical constants of amorphous silicon. J. Phys. E: Sci. Instrum. 16, 1214–1222 (1983).

