# Supplementary material for: Multiple-stable anisotropic magnetoresistance memory in antiferromagnetic MnTe
Source: Nat Commun. 2016 Jun 9;7:11623. doi: 10.1038/ncomms11623 (PMC4906165; doi:10.1038/ncomms11623)
Supplement: Supplementary Information — Supplementary Figures 1-12, Supplementary Table 1, Supplementary Notes 1-5 and Supplementary References [file ncomms11623-s1.pdf]

SUPPLEMENTARY NOTE 1: ANISOTROPIC MAGNETORESISTANCE PHENOMENOLOGY

In the main text we introduce anisotropic magnetoresistance (AMR) in analogy to ferromagnets where non-crystalline and crystalline contributions can be distinguished.[1,2] The non-crystalline component depends only on the angle subtended by magnetisation and current φ_M as

$$AMR_{\parallel} \propto \cos 2\varphi_M, \text{ and } AMR_{\perp} \propto \sin 2\varphi_M, \tag{1}$$

i.e., the signal is independent of the current flow direction with respect to the crystal. In polycrystalline samples with randomly oriented grains, this is therefore the only term contributing to the AMR. When normalised with the Hall bar aspect ratio (as in the definition of $AMR_{\parallel}$, $AMR_{\perp}$ in the main text), the non-crystalline AMR results in equal amplitudes of $AMR_{\parallel}$ and $AMR_{\perp}$. Note that this normalisation corresponds to the conversion of resistance R_{XX} into resistivity ρ_{xx}.

In single crystals, however, also other AMR terms beyond Supplementary Equation (1) can be present. They can be sub-classified into (pure) crystalline terms which depend only on the angle between magnetisation and some specific crystallographic direction and mixed terms which also depend on the current direction.[2] Each AMR term is characterised by an individual coefficient and these are known to be temperature-dependent (see for instance Fig. 2b in Ref. 2).

When analysing experimental data of $R_{\mathrm{XY}}(\varphi_B)$ and $R_{\mathrm{XX}}(\varphi_B)$ where φ_B is the angle between magnetic field $\mathbf{B}$ and current, it is important to realise that deviations from the $\sin 2\varphi_B$ ($\cos 2\varphi_B$) dependence do not automatically imply the presence of terms beyond Supplementary Equation 1. In ferromagnets, when the field is not sufficiently strong to overcome magnetocrystalline anisotropies, magnetisation may lag behind the field and $\varphi_B \neq \varphi_M$. This 'partial reorientation' of spin-axes distorts the $R_{\mathrm{XX}}(\varphi_B)$, $R_{\mathrm{XY}}(\varphi_B)$ signal so that it contains higher order terms (such as $\cos 4\varphi_B$) and/or it fails to have equal amplitudes of the longitudinal and transversal part even if Supplementary Equation (1) holds. A better way to isolate the crystalline terms is to use a Corbino disk geometry which averages all non-crystalline and mixed contributions due to multiple current directions with respect to the magnetisation (see Supplementary Note 4).

The difference between ferromagnets and antiferromagnets is that the magnetisation

aligns parallel to the saturating magnetic field in the former case while for antiferromagnets, the Néel vector tends to align to be perpendicular to **B** for sufficiently strong magnetic fields. Energy gain in this configuration is due to the Zeeman coupling to the net moment generated by the canting of the antiferromagnetic spin sublattices. In order to reach this perpendicular alignment in antiferromagnets one has to overcome the exchange-enhanced anisotropy energy, also called the spin flop field. All these considerations are limited to antiferromagnets with two collinear (antiferromagnetically coupled) magnetic sub-lattices of which MnTe is an example. In such systems the Néel vector orientation is defined by the direction of the magnetic moments.

SUPPLEMENTARY NOTE 2: MnTe THIN FILMS - GROWTH AND BASIC PROPERTIES

MnTe thin films were grown on single crystalline InP and SrF_2 substrates for transport and optical investigations, respectively. In both cases (111) oriented surfaces with indium and fluorine termination were used, respectively. Growth was performed by molecular beam epitaxy using elemental sources at a substrate temperature of 370 to 450°C. Using X-ray diffraction we find that the thin films grow in the hexagonal NiAs bulk phase (α-MnTe) and that no other phases are present (Supplementary Fig. 1a). In magnetometry measurements, although the signal is dominated by the temperature independent diamagnetism of the substrate, we are able to detect the transition temperature around ~ 300 K (Supplementary Fig. 1b) from a peak in the susceptibility. Furthermore we find that the magnetic moments are oriented in the sample plane as evident from the drop in susceptibility which is more pronounced for an in-plane magnetic field, equivalent to the report in Ref. 3.

The semiconducting properties of our α-MnTe thin films are clearly seen in the Hall measurement shown in Supplementary Fig. 1c, which shows a linear dependence of the Hall voltage on an out of plane applied magnetic field. We find a hole carrier density of $p = 6 \times 10^{18}$ cm^{-3} with a mobility of $\mu = 43$ cm^{2}V^{-1}s^{-1} at $T = 5$ K. The hole density found from the Hall measurements is increasing monotonously with temperature and reaches 4×10^{19} cm^{-3} at room temperature. Despite this monotonous increase of the hole density with temperature the longitudinal resistance of MnTe has a more elaborate temperature dependence as seen in Fig. 1d in the main text and explained by spin dependent scattering

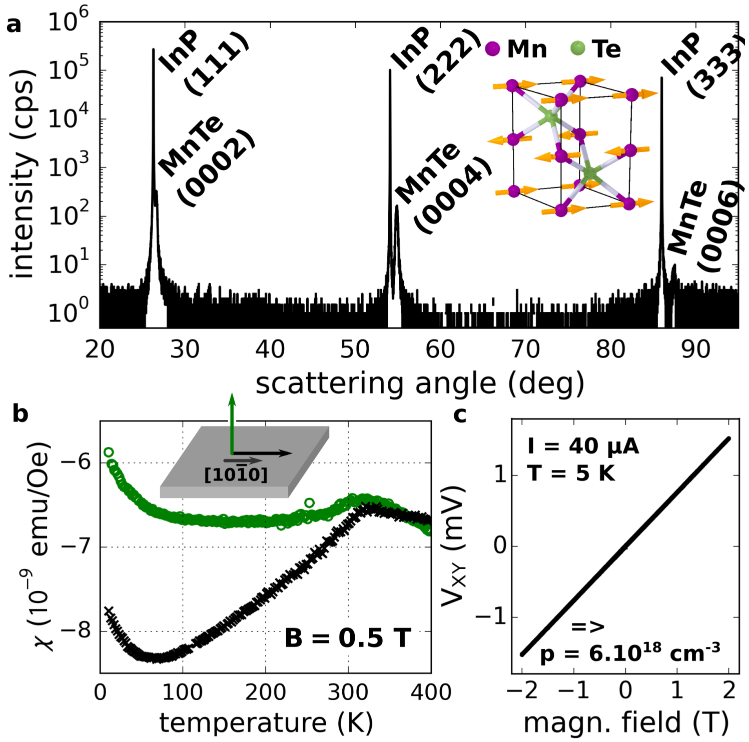

Supplementary Figure 1: **MnTe thin film properties.** a) Xray diffraction data of a 50 nm thick MnTe film grown on InP(111) showing only Bragg peaks of the hexagonal bulk α-MnTe phase which is sketched in the inset. b) Magnetic susceptibility χ of α-MnTe thin films measured for in-plane and out of plane field orientation in fields of 0.5 T. The drop of the susceptibility in the in-plane configuration is a result of the in-plane moment orientation. c) Hall measurement in order to determine the carrier density and mobility.

in literature[4].

Epitaxial orientation determined by X-ray diffraction

From transmission electron microscope images shown in Supplementary Fig. 2 we find that the films grow epitaxially oriented on both types of substrate. In combination with the X-ray diffraction reciprocal space maps shown in Supplementary Fig. 3 we determine the in-plane epitaxial orientation of the hexagonal α-MnTe lattice on the cubic substrates. We find the orientation of the c-planes (0001) is parallel to the (111) planes of the cubic substrate and the in-plane $[10\bar{1}0]$ direction of α-MnTe corresponds to the $[11\bar{2}]$ direction of the substrate. Note that we use the Miller-Bravais indices $(hkil)$ with $i = -h - k$ to denote

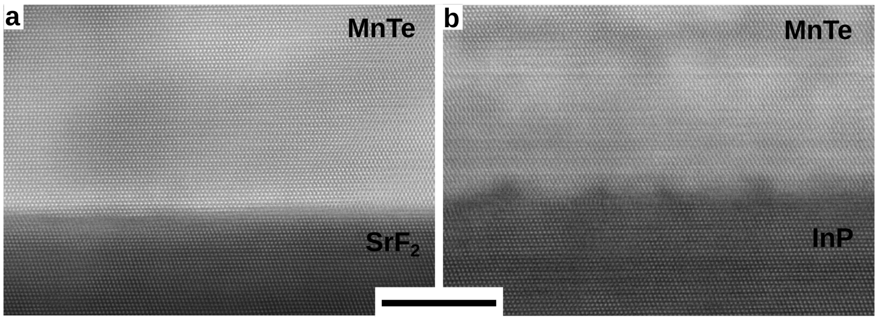

Supplementary Figure 2: **Cross-sectional scanning transmission electron microscope images of the MnTe/substrate interface.** Atomic resolution images of the MnTe/substrate interface show that for both a) SrF_2 and b) InP substrate epitaxial growth is obtained. Although the InP substrate interface shows higher roughness in both cases only c-axis oriented crystalline MnTe is found. The scale bar is 10 nm.

hexagonal directions and lattice planes. Even though the mismatch between α-MnTe and the InP(111) or SrF_2(111) substrates is below 1% the films grow relaxed for the investigated thickness range of 50 to 2000 nm. From the peak positions in the reciprocal space maps we find lattice parameters of $a = 4.1708$ Å $c = 6.6860$ Å for 50 nm MnTe grown on InP. With a mosaic block model[18] (dashed contour lines in Supplementary Fig. 3) we are able to describe the peak shape of the diffraction signal for both types of samples (grown on InP(111) or SrF_2(111)). Within such a model the peak shape of the various X-ray diffraction peaks is fitted simultaneously with the orientation distribution of the structural blocks. Both these parameters influence the peak shape and width and can be disentangled only by fitting various Bragg peaks as shown in Supplementary Fig. 3 simultaneously. For the sample used in the transport measurements (grown on InP) we find the lateral and vertical block size equal around 25 ± 5 nm and a Gaussian rotation distribution with standard deviation of 0.25 ± 0.1 degree was used for the simulations. The sample grown on SrF_2 can be described by blocks of same lateral size, however, with 50 ± 2 nm vertical block size and Gaussian rotation distribution with standard deviation of 0.15 ± 0.05 degree.

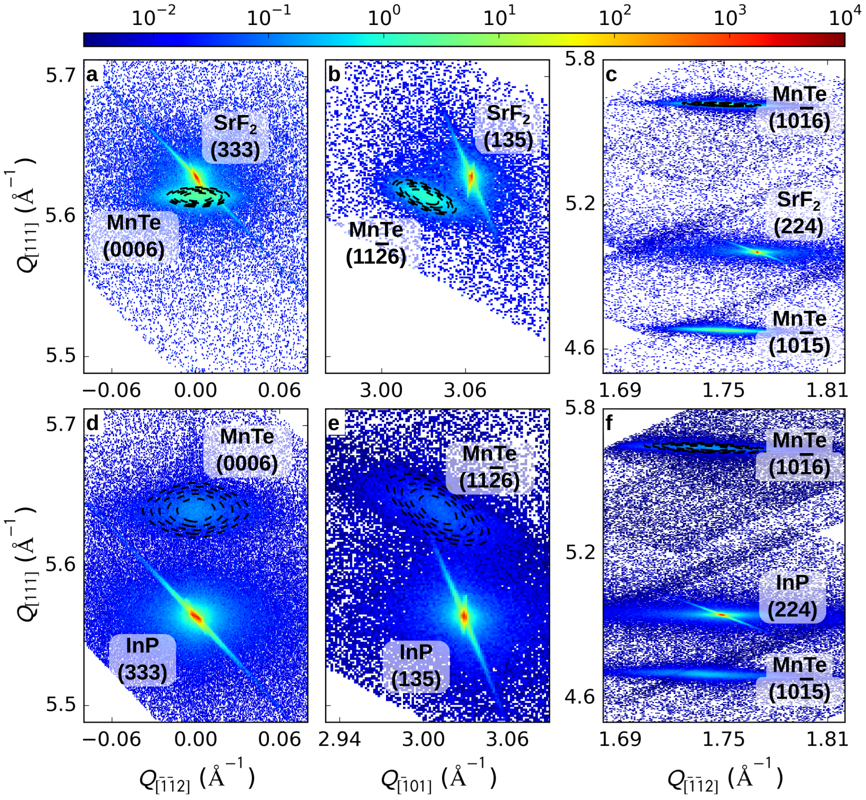

Supplementary Figure 3: **X-ray diffraction reciprocal space map measurements.** The color plot shows the experimental data with labeled peaks from the SrF_2/InP substrate and α-MnTe film. The shown measurements are for 200 nm MnTe on SrF_2 in panels (a-c) and 50 nm on InP in (d-f). Dashed contour lines mark the fit with the mosaic block model.

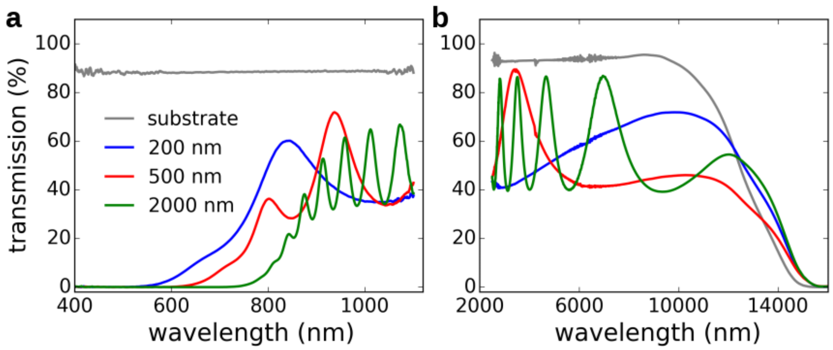

Supplementary Figure 4: **Optical characterization of MnTe by transmission measurements.** Transmission spectra of the α-MnTe thin films grown on $SrF_2(111)$ in the visible (left) and mid-infrared (right) spectral regions. Data for samples with various thicknesses allow us to extract a value of absorption coefficient shown in Fig. 1b in the main text.

SUPPLEMENTARY NOTE 3: MAGNETIC FIELD THRESHOLD AT ELEVATED TEMPERATURES

From the magnetometry data shown in the main text we conclude that the low temperature onset of a sizable magnetic moment occurs at a ~ 2 T in-plane field which may be associated with the onset spin-flop field in the antiferromagnetic domains. Supplementary Fig. 5 shows an enlarged view of the magnetic moment plotted in the main text Fig. 1c. Three different regions can be distinguished in the evolution of the magnetic moment under increasing field (applied inplane). Below 2 T their is no additional induced moment and the data follows the traces measured for field applied out of plane. Above the onset of spin flop at 2 T, a small magnetic moment appears. Due to the diversity of domains and the hexagonal symmetry, the spin flop does not occur over the entire sample at once and a transition region forms in which this moment develops. This region extends to approximately 5 T (at 5 K) above which the observed slope of $M(B)$ can be extrapolated to zero as it is common for spin flop transitions in other antiferromagnets.[5]

For higher temperatures the magnetometry measurements are shown in Supplementary Fig. 6a. These magnetometry measurements performed on 2000 nm thick MnTe films were also correlated with in-plane field sweeps in a Corbino geometry performed on 50 nm thick MnTe films and shown in Supplementary Fig. 6b. The longitudinal magnetoresistance shows a similar change of slope at low temperatures confirming the link between the variation of the transport data and the change of the magnetic response of the material. This further shows that the micromagnetic properties of thin and thick films are similar. The feature observed in the longitudinal resistance remains clearly visible at higher temperatures and allows us to conclude that the critical field needed for a reorientation of the moments decreases at higher temperatures. At 200 K the kink is sufficiently below 2 T to allow manipulation of the Néel vector by application of a 2 T field.

From inplane field rotations in the Corbino geometry, we can further estimate the size of the pure crystalline anisotropic magneto-resistance, which was found to be below 0.03% at 200 K and 2 T, while it is increased to 0.06% at 5 K and 2 T.

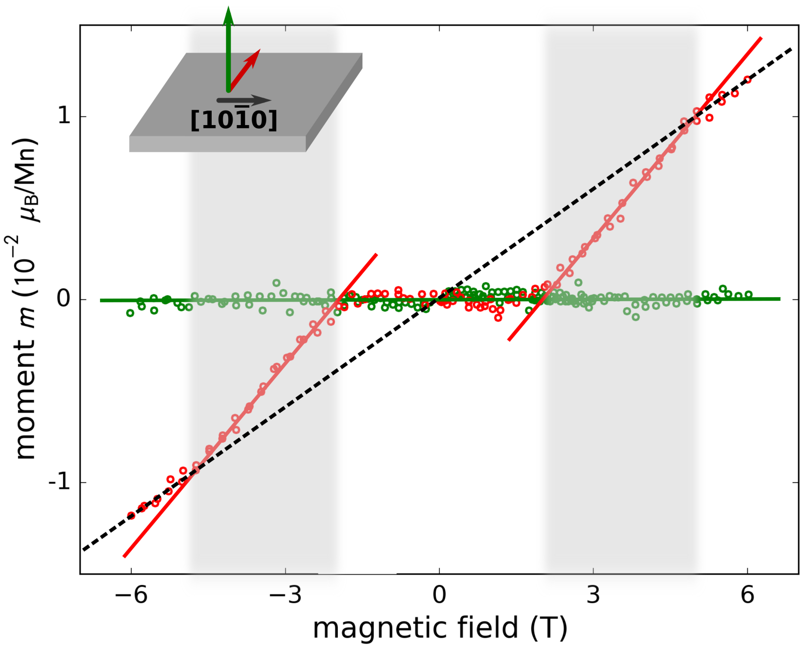

Supplementary Figure 5: **SQUID magnetic field sweeps for one inplane and the out of plane direction.** When applying an inplane magnetic field at low temperature we can observe three distinct phases in the evolution of the magnetic moment. 1) An initial phase with low susceptibility, which is rather similar independent of the direction of the field. 2) An onset of the development of magnetic moment around 2 T, 3) and a third phase where the extrapolation of the induced magnetic moment crosses zero. The slope of the out-of-plane measurement was subtracted from all the measured traces. The shown traces are equal to the respective curves in Fig. 1c in the main text and were recorded at 5 K.

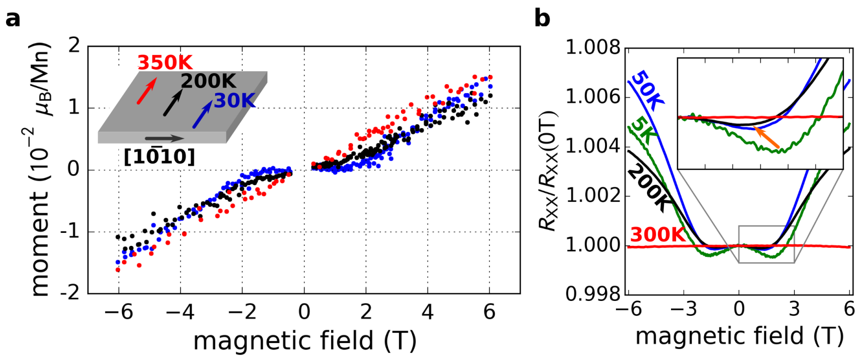

Supplementary Figure 6: **Magnetometry measurements and correlation with resistance at elevated temperatures.** a) Magnetic moment in in-plane magnetic field sweeps recorded at different temperatures. The temperature independent diamagnetic contribution of the substrate was subtracted as explained in the main text. The kink around 2 T gradually disappears at higher temperatures. b) Transport measurements in Corbino geometry at various temperatures show that an analogous kink can be observed in the resistance which via the crystalline AMR effect is sensitive to the orientation of the magnetic moments.

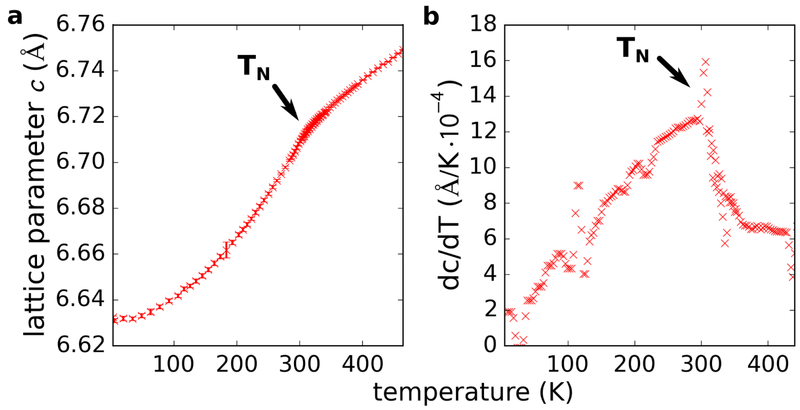

Supplementary Figure 7: **Temperature dependent c lattice parameter.** a) The out of plane lattice parameter shows an anomaly in its thermal expansion properties[6] which agrees with the AFM transition temperature found by the magnetometry measurements. b) In the derivative of the lattice parameter the anomaly is even more pronounced. Measurements were performed on a 2000 nm thick α-MnTe grown on InP. The Néel temperature T_N is indicated by arrows.

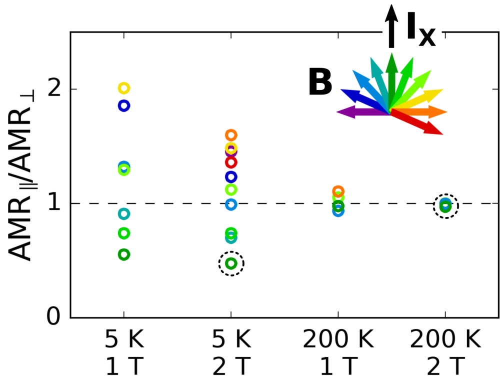

Supplementary Figure 8: **$AMR_{\parallel}$ to $AMR_{\perp}$ ratio for different field cooling directions.** The ratio of the AMR signal extracted from the longitudinal and transversal contacts shows strong dependence on the field cooling direction at 5 K. At 200 K and 2 T the ratio is independent of the history of the sample indicating that 2 T are above the critical field for reorientation of the moments. Dashed circles mark the data points extracted from the curves shown in Fig. 2a,b (main text).

SUPPLEMENTARY NOTE 4: STABILITY OF THE FROZEN STATES

In the main text we show the zero field AMR signal at 200 K (Fig. 2d) as it results after field cooling from above Néel temperature to 5 K. Supplementary Fig. 9a shows the equivalent data for different temperatures. The AMR signal is maximum at 150 K and reduces to zero at the magnetic order transition. To test the stability of this signal with respect to magnetic field at low temperature we performed field rotations at 5 K. Although the zero field AMR signal is decreased as can be seen in Supplementary Fig. 9b,c and 11 it can not be fully erased by neither 1 T nor 2 T rotations. Supplementary Fig. 11 shows the variation of the AMR signal of two memory states set by field cooling at angles $\varphi_{B,FC} = \pm 45°$ at 5 K tested by application of 0.5, 1.0, and 2.0 T fields. In agreement with Fig. 3 in the main text this figure shows how the zero field AMR memory states remain distinct after such a treatment. This is in contrast to equivalent rotations at 200 K, where a rotation of a 2 T field (above the spin-flop field) is able to destroy the frozen state as seen in main text Fig. 3d.

The fact that with 2 T at 200 K we are in a different regime as at 5 K is also seen in the ratio of the $AMR_{\parallel}$ to $AMR_{\perp}$ amplitudes shown in Supplementary Fig. 8. Only at 200 K and 2 T the respective amplitudes are equal and the ratio is independent of the device history. In the other cases (5 K: 1 and 2 T, and 200 K 1 T) the data show a dependence on the field cooling direction and strongly deviate from the ideal ratio of 1. We ascribe this to only partial rotation of the magnetic moments as explained in Supplementary Note 1.

Due to the fact that at 200 K we are able to overcome the spin-flop field with a 2 T in-plane field we are also able to perform magneto-recording when cooling in field from 200 to 5 K. Corresponding data are shown in Supplementary Fig. 10 and clearly demonstrate that at 200 K AMR traces are found in zero field although the Néel temperature was never overcome.

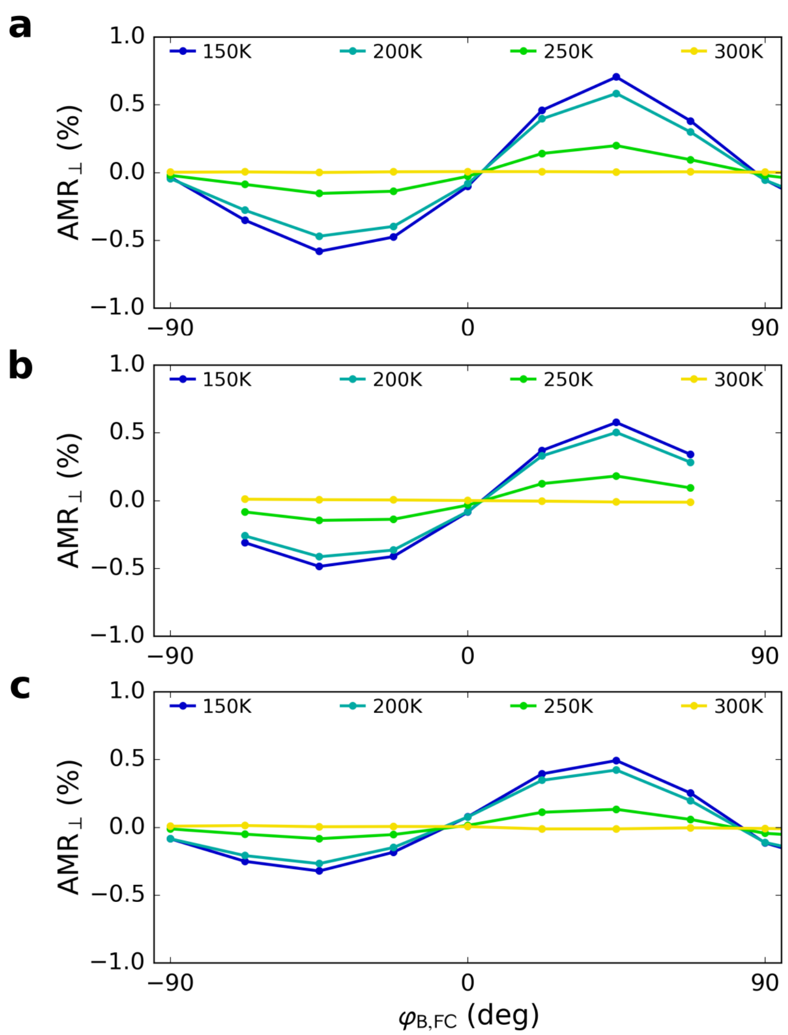

Supplementary Figure 9: **Stability of the AMR signal against field rotations.** a) The zero field AMR signal induced by field cooling in various directions is shown for different temperatures. Below 300 K there is clear semi harmonic signal due to the frozen moment orientation. The same signal with equal symmetry is found in a measurement after the magnetic field of b) 1 T and c) 2 T magnitude was rotated in three perpendicular planes at 5 K. Although the amplitude of the AMR is slightly reduced these field rotations can not destroy the frozen state.

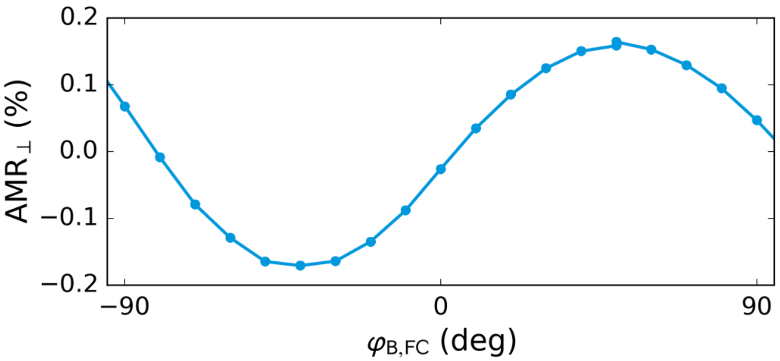

Supplementary Figure 10: **Magneto-recording by cooling from 200 to 5 K.** $AMR_\perp$ data recorded in zero field at 200 K after field cooling from 200 to 5 K in a 2 T applied field.

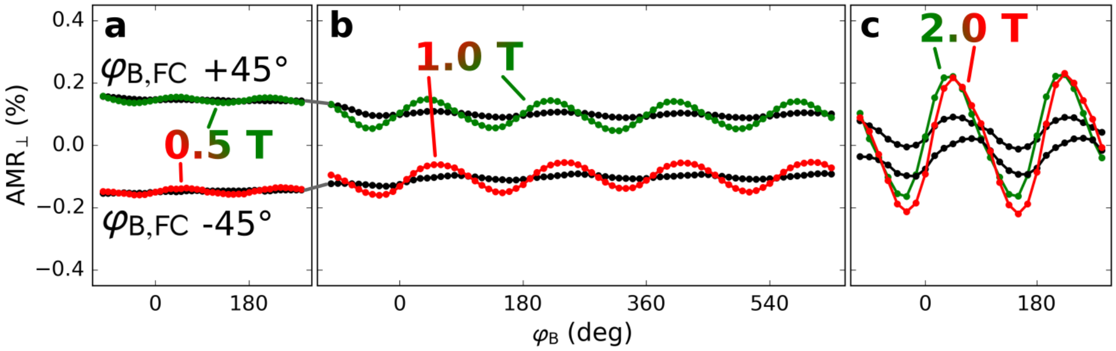

Supplementary Figure 11: **Stability of the zero field AMR against magnetic field.** The stability of two memory states set by the heat-assisted magneto-recording from 350 K with a 2 T writing field applied at angles $\varphi_{B,FC} = \pm 45°$ tested at 5 K by a rotating a) 0.5 T, b) 1.0 T, and c) 2.0 T fields. Red/green curves correspond to the read-out resistance measurements with the field on while black lines are obtain after removing the perturbing field at each φ_B. Finally after the 2 T rotation black zero field AMR signal is reduced but clearly non-zero. Panel b) is equivalent to Fig. 3e in the main text.

SUPPLEMENTARY NOTE 5: MULTIPLE-STABILITY AND ANISTROPIC MAGNETORESISTENCE MODEL

In order to describe the domain population after the field cooling and the resulting AMR signal we use the Stoner–Wohlfarth model. We apply it to each magnetic sub-lattice of α-MnTe, take into account the magnetocrystalline anisotropy of the material and the exchange energy which describes the mutual interaction of the sub-lattices. We restrict ourself to a 2D description which is sufficient to describe the field cooling in different in-plane magnetic fields. Every magnetic domain consists of two equal ferromagnetic sub-lattices which have opposing magnetisation vectors $\mathbf{M}_{1,2}$. When a magnetic field $\mathbf{B}$ is applied in the plane of the sample the magnetic moments may cant with respect to each other and thereby tilt away from the easy axis direction. The relevant angles are defined with respect to the current or easy axis direction as sketched in Supplementary Fig. 12.

The total energy E per sample volume V of a single domain is given by the sum of exchange, Zeeman, and magneto–crystalline anisotropy energies:

$$E/V = J_{\text{ex}}\hat{\mathbf{M}}_1 \cdot \hat{\mathbf{M}}_2 - \mathbf{B} \cdot (\mathbf{M}_1 + \mathbf{M}_2) + E_{\text{MAE}}(\hat{\mathbf{M}}_1) + E_{\text{MAE}}(\mathbf{M}_2) \tag{2}$$

where J_{ex} is the exchange constant, E_{MAE} is the magneto-crystalline anisotropy function, and a hat (" ˆ ") denotes a unit vector. Using the angles defined in Supplementary Fig. 12 and the exchange field defined as $B_{\text{ex}} = J_{\text{ex}}/M$ with $M = |\mathbf{M}_{1,2}|$ this can be rewritten as

$$\begin{aligned} E/V = &- MB_{\text{ex}} \cos(\psi_1 + \psi_2) - MB \left[\cos\left(\varphi_{\text{B}} - (\alpha + \psi_1)\right) - \cos\left(\varphi_{\text{B}} - (\alpha - \psi_2)\right)\right] \\ &+ E_{\text{MAE}}(\psi_1) + E_{\text{MAE}}(\psi_2). \end{aligned} \tag{3}$$

The exchange field is estimated from the Néel temperature as $B_{\text{ex}} = k_{\text{B}}T_{\text{N}}/\mu_{\text{B}}$, where we use the Boltzmann constant k_{B} and the Bohr magneton μ_{B}. The magneto-crystalline anisotropy energy density is expressed as

$$E_{\text{MAE}}(\psi) = K_{\text{MAE}} \sin^2 3\psi, \tag{4}$$

following from the six-fold in-plane symmetry of the hexagonal material. Here, K_{MAE} has units of energy density and can be obtained from the spin flop field B_{SF} and the exchange field by $K_{\text{MAE}} = 12MB_{\text{SF}}^2/B_{\text{ex}}$.

In order to determine the population of the three different easy axes, we calculate the energy of these domains when a field is applied at a given angle. Independently for every

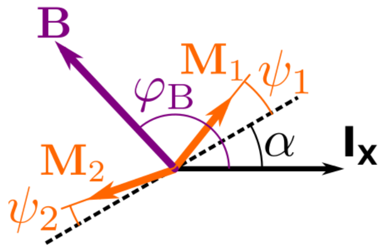

Supplementary Figure 12: **Sketch of the parameters in the Stoner–Wohlfarth model.** The angles of the magnetic moments of the two sub-lattices ($\mathbf{M}_{1,2}$) deviate slightly from the easy axis direction (dashed line) upon the application of magnetic field $\mathbf{B}$. The angles relevant for the modelling are indicated.

domain we determine the orientation of the moment by minimizing the energy given by Supplementary Equation 3. Using the difference of the domain energy with respect to the zero field (ΔE) case we can calculate the population of the easy axis using the Boltzmann statistics. The freezing occurs at a temperature T_F which enters in the calculation of the relative population w_i of the different domains:

$$w_i = \frac{e^{\Delta E_i/(k_\mathrm{B}T_\mathrm{F})}}{\sum_{j=1}^{3} e^{\Delta E_j/(k_\mathrm{B}T_\mathrm{F})}} \tag{5}$$

We use the Néel temperature for T_F. Note that it is at this point where the domain volume enters since total energies need to be obtained from Supplementary Equation 3. We use the diameter d of spherical domains to describe their volume since spherical domains were also successfully used in the mosaic block model described earlier. The population numbers w_i are used to calculate the transversal and longitudinal zero field AMR signal which is proportional to $\sum_{i=1}^{3} w_i \sin 2\alpha_i$ and $\sum_{i=1}^{3} w_i \cos 2\alpha_i$, respectively (assuming that non-crystalline AMR is dominant, see Supplementary Notes 1 and 4).

It is possible to see from the calculated zero field AMR for different domain sizes d how the field cooling efficiency is increasing for bigger domains (it leads to a stronger AMR; see Fig. 4c in the main text). When the domains get so large that almost only one domain type is populated the AMR signal is becoming step-like. The results of our calculations compared to the experimental data, which show a transitional behavior between the harmonic AMR and the step-like signal, are shown in Fig. 2d of the main text. By performing least squares optimization of the domain size, easy axis direction and the amplitude of the AMR we were able to achieve a nearly perfect agreement with the experimental data. Supplementary

Supplementary Table I: Model parameters used for the calculations in the main text. The exchange energy was extracted from the Néel temperature. The magnetic moment from neutron diffraction data in Ref. 7 was used.

B_{ex}	B_{SF}	T_{F}	$\lvert M_{1,2}\rvert$	easy axis direction	d	AMR ampl.
450 T	2 T	T_{N}	4.76 μ_{B}	$\langle 10\bar{1}0\rangle$	20 ± 5 nm	$1.3 \pm 0.2\%$

Table I summarizes the model parameters used.

The errors of these three optimized model parameters are estimated not only from the numerical errors that follow from the least squares optimization but also considering correlations with the other fixed parameters. We performed the fitting considering variations in the exchange energy, anistropy energy and magnetic moment by 50% of the value given in Supplementary Table I and found that the easy axis direction implied is along the $\langle 10\bar{1}0\rangle$ directions in all cases. The domain size was found to vary around 20 nm by approximately 5 nm which is similar to the domain size determined by the mosaic block model. The saturated AMR amplitude at 200 K is found to be $1.3 \pm 0.2\%$. Using the very same parameters the variation of the AMR amplitude with field cooling strength was calculated and found to qualitatively agree with the experimental observations.

[1] McGuire, T. & Potter, R. Anisotropic magnetoresistance in ferromagnetic 3d alloys. *IEEE Trans. Magn.* **11**, 1018–1038 (1975).

[2] Rushforth, A. W. *et al.* Anisotropic Magnetoresistance Components in (Ga,Mn)As. *Phys. Rev. Lett.* **99**, 147207 (2007). URL {http://link.aps.org/doi/10.1103/PhysRevLett.99.147207}.

[3] Madelung, O., Rössler, U. & Schulz, M. *MnTe: crystal structure, physical properties*, vol. 41D (SpringerMaterials, Springer-Verlag GmbH, Heidelberg, 2000).

[4] Magnin, Y. & Diep, H. T. Monte Carlo study of magnetic resistivity in semiconducting MnTe. *Phys. Rev. B* **85**, 184413 (2012).

[5] Jacobs, I. S. Spin-Flopping in MnF2 by High Magnetic Fields. *J. Appl. Phys.* **32**, S61–S62 (1961).

[6] Greenwald, S. The antiferromagnetic structure deformations in CoO and MnTe. *Acta Crystallogr.* **6**, 396–398 (1953).

[7] Szuszkiewicz, W., Hennion, B., Witkowska, B., Usakowska, E. & Mycielski, A. Neutron scattering study of structural and magnetic properties of hexagonal MnTe. *Phys. Stat. Sol. C* **2**, 1141–1146 (2005).
