# Supplementary material for: Multiple-stable anisotropic magnetoresistance memory in antiferromagnetic MnTe
Source: Nat Commun. 2016 Jun 9;7:11623. doi: 10.1038/ncomms11623 (PMC4906165; doi:10.1038/ncomms11623)
Supplement: Peer Review file [file ncomms11623-s2.pdf]

Editorial Note: this manuscript has been previously reviewed at another journal that is not operating a transparent peer review scheme. This document only contains reviewer comments and rebuttal letters for versions considered at Nature Communications.

Reviewer #1 (Remarks to the Author)

Authors responded to my comments and questions satisfactorily. I would like to recommend the publication in Nature Communications.

Authors response:
We thank the referees for their constructive feedback during the review process.
